# Object recognition ability predicts episodic location memory, enhanced by meaningfulness

**DOI:** 10.1007/s00426-026-02248-y

**Published:** 2026-02-07

**Authors:** Nurit Gronau, Conor J. R. Smithson, Isabel Gauthier

**Affiliations:** 1 Education and Psychology Department, The Open University of Israel, Raanana, Israel; 2 Department of Psychology, Vanderbilt University, Nashville, TN USA

## Abstract

People differ in their ability to distinguish visually similar items, a domain-general ability known as *o* Richler (Psychological Review 126:226–251, 2019). While *o* typically involves extracting invariant object properties, we investigated whether it also relates to long-term memory for episodic information extrinsic to object identity, specifically, object location. We further examined whether this relationship is influenced by stimulus meaningfulness, a factor known to enhance long-term memory, by using both high- and low-meaning stimuli. Participants completed a location memory test, a series of visual object-recognition tasks assessing *o*, and other cognitive covariate measures. Results showed a positive correlation between *o* and location memory, which was stronger for high-meaning than for low-meaning stimuli. This suggests that semantic content may enhance the link between object recognition and episodic location memory. Importantly, these effects remained after controlling for age, gender, low-level visual perception, working memory, and general intelligence. Our findings indicate that domain-general object recognition ability contributes to episodic memory by supporting the binding of meaningful objects to their spatial context. This challenges traditional cognitive boundaries by integrating current knowledge about individual differences in perception and memory, with semantic meaning acting as a significant moderator.

**Supplementary Information:**

The online version contains supplementary material available at 10.1007/s00426-026-02248-y.

## Introduction

Domain-general object recognition ability, or *o*, explains shared variance in performance across different object recognition tasks that span multiple domains (Richler et al., 2019; Smithson & Gauthier, 2026; Sunday et al., 2021). At its core, *o* reflects the ability to distinguish visually similar objects, such as differentiating between two bird species at the subordinate level (Richler et al., 2019). It is, however, usually measured with novel objects so that semantic knowledge and individual experience with familiar categories do not complicate its interpretation (Richler et al., 2017; Smithson et al., 2024). As such, an important characteristic of *o* is its domain-generality: an *o* factor measured with several categories of familiar objects (e.g., birds and planes) correlates perfectly at the latent level with an *o* factor measured using novel, unfamiliar objects (Sunday et al., 2021). This consistency across familiar and novel stimuli suggests that *o* represents a general perceptual ability rather than accumulated expertise.

In recent work, we found that *o* can be measured using tasks that vary substantially in their cognitive demands - some tasks primarily stress perception whereas others make substantial memory demands. For example, perceptual tasks require online recognition of visually similar objects across changes in viewpoint or noise. Memory-based tasks focus on object encoding and maintenance, followed by forced-choice recognition among highly similar exemplars. Importantly, most memory tests center on memorization of intrinsic shape features, as success on these tasks requires making fine-grained shape-feature distinctions. Although previous studies have demonstrated that people can retain quite precisely the perceptual details of real-world objects (Brady et al., 2008; Konkle et al., 2010), memorizing the fine details of novel objects proves to be a much more challenging task, yielding dramatically poorer overall visual recognition rates (e.g., Shoval et al., 2023a). A high-*o* person, nevertheless, may represent an unfamiliar stimulus with rich detail, allowing them to subsequently make fine subordinate-level discriminations during the memory test. Thus, despite differences in their nature, both perceptual and memory-based tasks used to measure *o* tap a common underlying object recognition ability (Smithson & Gauthier, 2025). The memory demands in the measurement of *o*, therefore, are incidental to the core ability, which involves distinguishing visually similar objects and shapes, regardless of task format.

Because success on *o* tasks depends on identifying diagnostic features across changes in viewpoint or noise, *o* ability is often assumed to rely on invariant representations that abstract away instance-specific details. For instance, emphasizing invariance would allow someone to recognize a warbler regardless of its pose or lighting conditions, focusing on identity-relevant features while ignoring contextual variations. This raises questions regarding the fate of such contextual details during online perception and subsequent memory: Are these details genuinely discarded, or might their retention *depend* on the precision with which intrinsic object features are encoded? If so, should a bird watcher higher in *o* also be better able to remember episodic information associated with the warbler? More broadly, this possibility suggests a link between memory for intrinsic object features and memory for extrinsic, episode-defining properties. This question becomes particularly intriguing when considering incidental, often arbitrary contextual details, such as the specific orientation of the warbler, or the branch on which it happens to be resting. While the skills of visual object recognition and episodic memory are typically studied separately, both abilities are useful, and may interact in many real-world applications. For instance, a crime scene investigator must quickly identify objects despite occlusions but also remember their exact placement, even when the room has been left in a disorganized state. Similarly, a field biologist needs to recognize animal tracks regardless of substrate conditions but also remember the specific timing and location of each sighting. This raises the possibility that individuals who form richer and more precise intrinsic object representations may provide a stronger foundation for binding those items to their episodic contexts.

Here, we explore for the first time whether *o* relates to long-term memory for a property extrinsic to object shape or identity, specifically its location - a key facet of episodic memory (Ranganath, 2010; Tulving, 1972). Although the theoretical definition of *o* does not directly predict whether it should support better episodic memory for non-shape features, addressing this question is important for integrating current knowledge about individual differences in perception and their potential relationship with memory. Moreover, as explained above, *o* is thought to reflect perceptual skills that do not require linkage to semantic knowledge, which is why it can be estimated using novel objects. Yet in the real world, the semantic meaning of objects often supports visual perception through increased familiarity and/or top-down knowledge. While the processing of both shape and location primarily relies on perceptual abilities, the richer categorical and subcategorical knowledge that presumably characterizes high-*o* individuals can penetrate early stages of visual analysis and modulate object representations (Collins & Olson, 2014; Goldstone, 1994). In addition, semantic information can alter how visual experts weigh and selectively attend to subtle perceptual features (Gauthier et al., 2003; Johnson & Mervis, 1997). At the same time, stored semantic knowledge affects episodic memory, as the retrieval of past events depends in part on reinstating the conceptual representations that were active during encoding (Renoult et al., 2019). We therefore further examine whether, and how, the semantic meaning of real-world objects potentially interacts with high perceptual ability (*o*) to influence location memory. To this end, individuals with varying *o* levels encoded objects that were either low or high in meaningfulness and later reported each object’s location.

Our first prediction is that location memory will be better for objects that are more meaningful. As mentioned above, our study included two kinds of objects, low- and high-meaning objects, as defined by independent ratings (Shoval et al., 2023b, see details below). Prior work found that both object identity and their arbitrary location are better remembered in long-term memory when items are more meaningful (Gronau et al., 2024; see also Taevs et al., 2010; DeWitt et al., 2012). This effect has been attributed to a ‘Resource-limited’ account, whereby more familiar items are *subjectively* perceived as less complex and thus consume fewer limited working-memory resources. In other words, the streamlined encoding of such stimuli allows more capacity to be devoted to extrinsic details and to their binding to the object in long-term memory (Popov & Reder, 2020; Zhang et al., 2020). We expect to replicate this effect, although we are not directly investigating its underlying mechanism.

Our second prediction is that people with a higher *o* will have a better memory for the location in which objects were studied, beyond individual differences in general intelligence, working memory and other factors such as age or gender. Although spatial position is extrinsic to an item’s core identity and features, object location and identity are not perceived as strictly independent. For instance, objects shown in the same location are more likely to be reported as having the same identity (Babu et al., 2023; Golomb et al., 2014), and splitting attention across different locations disrupts feature binding (Dowd & Golomb, 2019).

One reason that high-*o* individuals may better retrieve the locations associated with studied objects is that streamlined object processing during encoding preserves capacity for registering context-related details, again consistent with resource-limited accounts (e.g., Popov & Reder, 2020). Indeed, individuals who benefit from enhanced perceptual encoding, for instance through perceptual expertise, show a working memory advantage for items within their domain of expertise (Curby et al., 2009). Likewise, stronger object representations in those with a higher *o* may allow for more item-context binding. We acknowledge, however, that this is not the only possible reason *o* may predict location memory. Another explanation comes from a retrieval-gating account, according to which episodic memory retrieval occurs when a retrieval cue sufficiently overlaps with a stored memory representation, leading to the reactivation of the encoded information (Tulving & Thomson, 1973). In our task, memory for an object’s location depends on, or is ‘gated’ by, the ability to recover the object representation itself (particularly because each location is associated with multiple different objects). In other words, the objects serve as cues: if an object is not remembered at least in part, its associated location cannot be retrieved. In that case, more object locations should be retrieved accurately as a function of *o* because the objects themselves are better remembered. Thus, both a resource-limited account and a retrieval-gating account make the same prediction, contrary to a common null hypothesis that *o* will not be related to memory for location since it reflects an ability specific to the invariant processing of shape information. Note, however, that the limitations of our correlational approach allow us to ask, for the first time, *whether o* can predict location memory, not *why* it does.

**Fig. 1:**
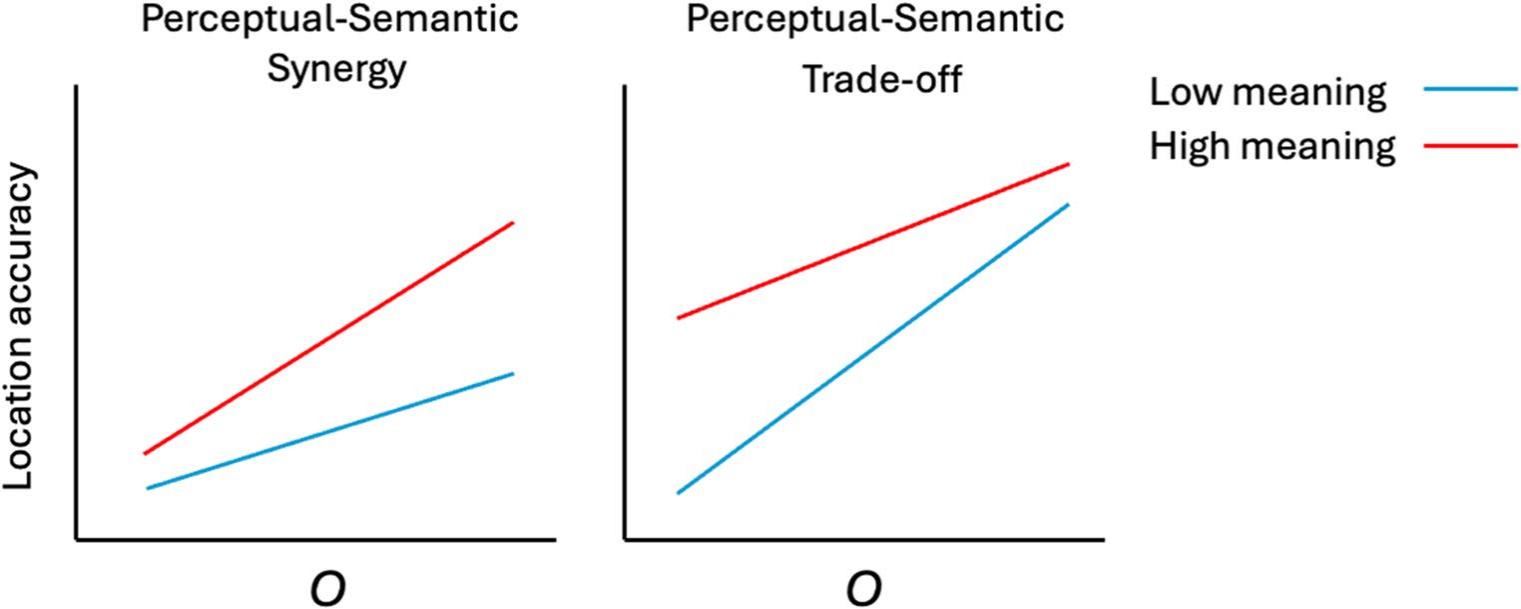
Two different predictions for how individual differences in *o* and object meaningfulness interact to predict location accuracy

We have predicted two main effects: better location memory for meaningful objects and better location memory with a higher *o*. Our third and most critical prediction, however, concerns the role of semantic knowledge in mediating a possible perception-memory relationship. Here, two different hypotheses can be formulated. The first is that perceptual abilities (indexed by *o*) can fuel a self-reinforcing cycle that enhances memory (and eventually may shape expertise development in a specific field). We call this the Perceptual-Semantic Synergy account. For example, domain-specific episodic memories (e.g., a birder remembering a warbler’s pose in morning light) could provide foundational material that, through abstraction and integration, builds semantic knowledge and supports expertise (Greenberg & Verfaellie, 2010; Tulving, 1972). Richer semantic knowledge can, in turn, improve episodic recall (e.g., Renoult et al., 2019), as when knowledge of typical warbler behavior supports memory of its specific pose or the particular branch it was perched on. This has important implications for the interaction between object meaning and *o*: High-*o* individuals encode objects with precision, automatically, and even when object shape is task-irrelevant (McGugin et al., 2022). By definition, high-*o* individuals are better at subordinate-level discriminations (Sunday et al., 2021). Therefore, in our study, high-*o* people should encode both low- and high-meaning objects more precisely, but a precise representation of a high-meaning object is likely to lead to categorization that is more specific (for instance, a chair may be recognized as a “colonial-style antique chair”). This can unlock access to a wider associative network of semantic information (e.g., this style prioritizes practicality, it is often put together with wooden pegs rather than nails…), thereby increasing meaningfulness. In other words, a familiar object encoded with more visual precision gains richer semantic content as well. To the extent that prior meaningfulness has been found to increase episodic memory for objects, including for their location (Gronau et al., 2024; Taevs et al., 2010), this suggests that *o* would be positively correlated with the magnitude of this effect. In other words, the Perceptual-Semantic Synergy account predicts a stronger positive correlation between *o* and the accuracy of location judgments for high-meaning relative to low-meaning objects (Fig. 1, left).

An alternative hypothesis posits an interaction between *o* and object meaning in the opposite direction. Objects in our study belong to different basic levels. In the high-meaning condition, this allows items to be largely remembered using verbal and semantic information in addition to visual information. Note that the availability of unique verbal labels does not mean that visual information is not encoded, as demonstrated in work where people encode massive quantities of such objects and can later discriminate them from highly similar objects sharing the same verbal code (Brady et al., 2008). In the low-meaning condition, however, the memorization of objects must primarily rely on visual information because items are less familiar and nameable. We could therefore expect that perceptual skills may be particularly critical for low-meaning objects, resulting in a stronger correlation with *o* ability than for high-meaning objects. This prediction (Fig. 1, right) assumes that perceptual and semantic information function separately but can trade off, such that people rely more on one when the other is absent. We call this the Perceptual-Semantic Trade-off account. Luckily, while both of these hypotheses predict removable interactions (Wagenmakers et al., 2012), each one presents a qualitative contrast with the other. This means that support in favor of one hypothesis provides strong evidence against the other.

It is worth noting that, theoretically, a third possibility exists: since *o* is a perceptual ability in its essence, it might not interact with the semantic meaning of stimuli in predicting location memory. However, we consider this scenario to be rather unlikely, as a sharp perception-memory distinction may reflect historical conventions more than cognitive reality (Firestone & Scholl, 2016).

The competing predictions outlined above have never been directly tested. If *o* predicts memory for where an object was experienced - not just what it looks like- it would significantly advance our understanding of the relationship between perception and memory. How semantic meaning interacts with *o* in predicting location memory will further constrain our understanding of this relationship.

## Method

All experimental materials and results are available at the Open Science Framework and can be accessed at https://osf.io/hy763/?view_only=be6bc532f4fa490d98e81e7eb1d8eb86. This study was not preregistered. All experiments were performed in line with the principles of the Declaration of Helsinki. Approval was granted by the Ethical committee of the Open University of Israel (#3668) and of Vanderbilt Institutional Review Board (#222116).

### Participants

We recruited participants from a prior study in which we had collected measures of object recognition and other cognitive covariates. The original study (Smithson & Gauthier, 2025) was conducted on the Prolific.com platform and recruited participants residing within the US, 18 to 45 years old, reporting fluency in English and normal or corrected-to-normal vision, and having a > 95% approval rating for past studies. That study was 2 sessions long, with 333 participants completing session 1, 298 completing session 2 and 275 people passing exclusion criterion (i.e., not performing below chance on more than one test). We invited 255 of these participants (excluding those who were at chance on more than 2 of the 14 tasks in the original studies) for a new task, approximately 1 month after completion of the Smithson et al. (2025) study. Both studies could only be completed on a computer screen, not a mobile or tablet. We were able to collect data from 159 participants, and 154 of them performed above chance (25%) on the 4-alternative forced choice episodic location task. Their mean age was 33.8 (SD = 6.6), and they self-reported gender as follows: 83 women, 72 men, 1 prefer not to say. A sensitivity analysis for detecting the incremental contribution of *o* (after accounting for covariates) indicated that with *N* = 154, α = 0.05, and power = 0.80, the smallest effect size detectable at this level of power is an incremental effect for *o* of R² = 0.049 (this is the same for 6 and 7 predictors, so it applies to all our multiple regressions). Informed consent was obtained from all individual participants included in the study.

### Procedure

#### Tasks from Smithson and Gauthier (2025)

Eight tasks measured participants’ object recognition skills and their proficiency in distinguishing highly similar visual shapes – tasks that when combined form one’s *o* measure. Four of these tasks were based on increased perceptual demands (e.g., rapid presentation time, noise on the images, degraded silhouettes), while the remaining four relied primarily on memory demands (e.g., encoding a large number of objects, temporal delays, and requiring associations between objects). Four additional tasks measured low level visual ability using oddball decisions with simple visual features, three tasks were included to measure general intelligence (*g*), and 2 tasks were included to measure working memory. We summarize these tasks below and details can be obtained in the original work. Eighteen simple attention checks were embedded throughout the test battery to assess engagement and data quality. These checks typically required straightforward responses to obvious stimuli or explicit instructions (e.g., “click the leftmost option”). Participants were excluded if they made more than 1 attention error.

The specific control measures were not selected explicitly for the present work but rather were chosen by Smithson and Gauthier (2025) because they aimed to demonstrate that *o* could be measured with tasks that also tapped working memory and general intelligence, or tapped low level vision and general intelligence. Working memory and intelligence are powerful sources of variability in most cognitive tasks, as may be low level vision for many visual tasks, so together they provide a strong control of variability that is not specific to *o*.

**Fig. 2:**
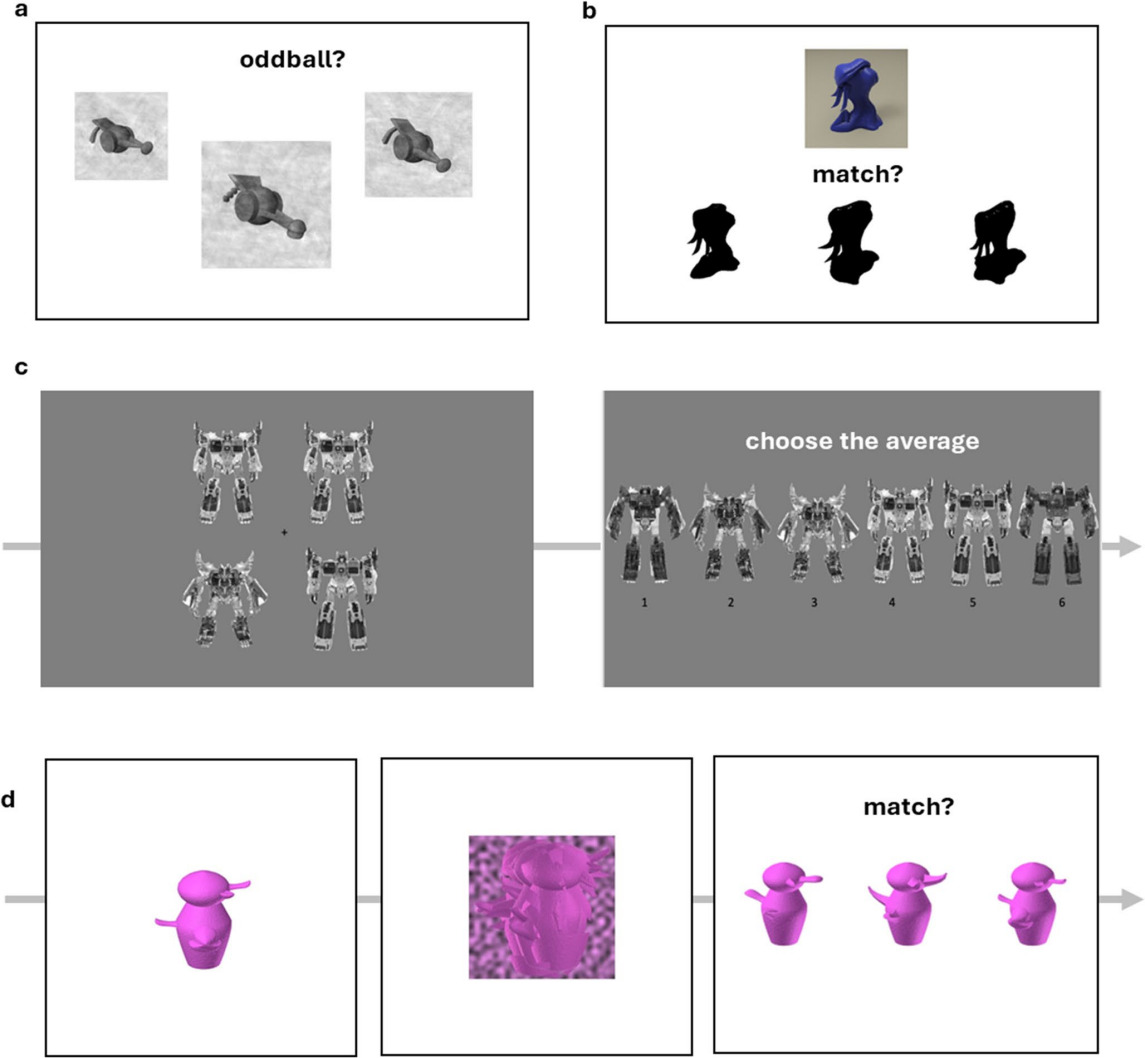
Example trials from (**a**) the Many Objects Oddball task; **b** the Silhouette Masking task; **c** the Ensemble Perception with Transformers task and (**d**) the 3AFC Matching with Asymmetrical Greebles task. Performance on these 4 tasks from Smithson and Gauthier (2025) was aggregated into an Op score

*Perceptual* *o* *tasks. *Four tasks emphasized perceptual discrimination under challenging viewing conditions (Fig. 2). *Many Objects Oddball* required identifying which of three simultaneously presented objects differed in identity across 45 trials. Two objects shared identity but varied in size and orientation, while the third was slightly different. Objects appeared for 750–4000 ms and participants clicked the location where the odd object had appeared. Novel object categories changed each trial, with feedback provided after responses. *Silhouette Matching* involved matching target objects (YUFOs) to one of three simultaneously presented silhouettes across 40 trials, often requiring viewpoint invariance when silhouettes appeared rotated relative to targets. *Ensemble Perception with Transformers* tested averaging ability across 50 trials where participants viewed four robot figures (1 s), then selected which of six morphs best represented their average. Performance was measured as absolute error in degrees within the circular morph space. *3AFC Matching with Asymmetrical Greebles* required matching target objects after a 500 ms visual mask across 51 trials. Four blocks systematically varied target presentation times (300–1000 ms), viewpoints (same vs. ~30° horizontal rotations), and noise levels.

**Fig. 3:**
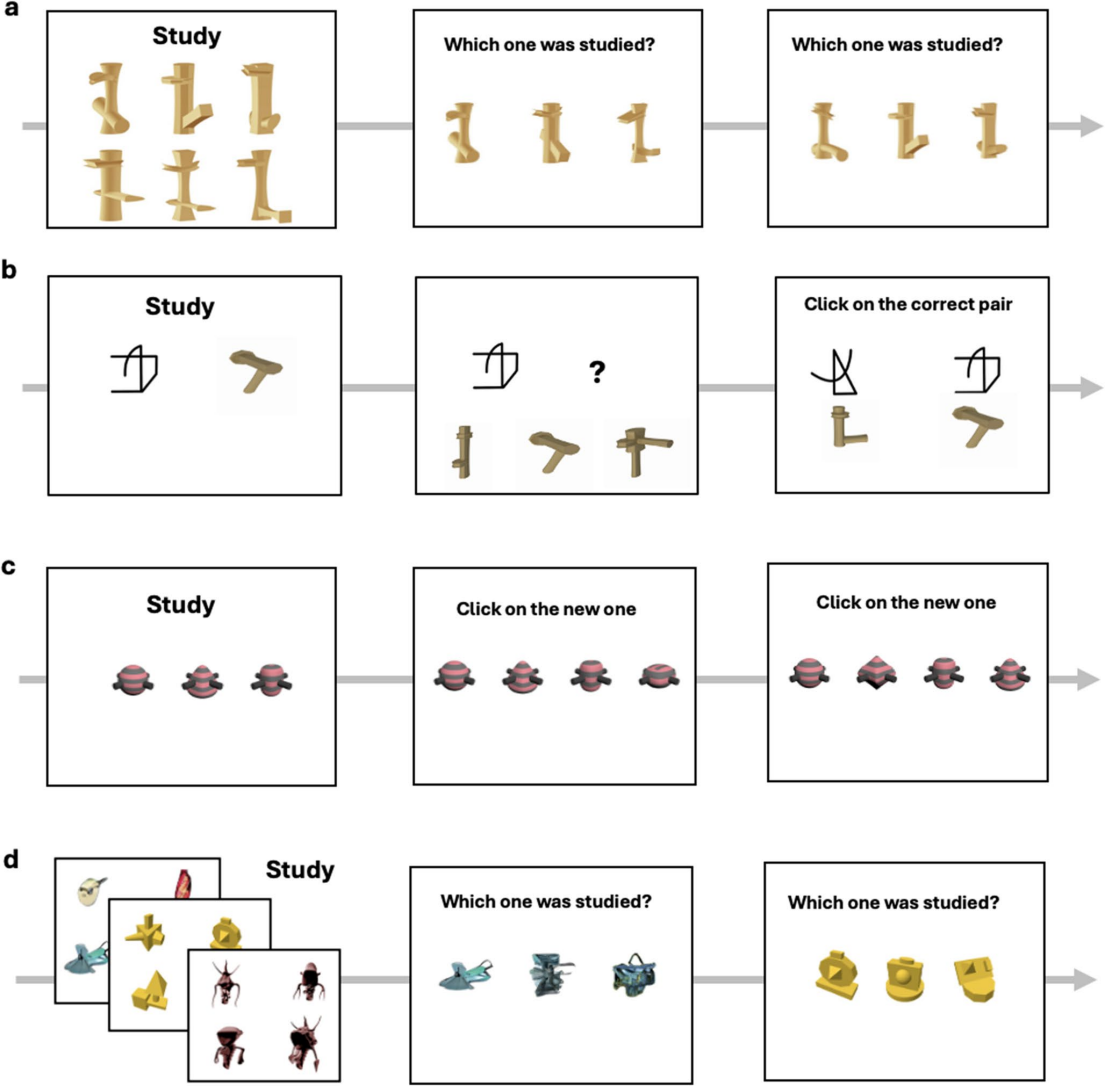
Example trials from (**a**) the Learning Exemplars with Vertical Ziggerins task; **b** the Pairs Objects task; **c** The New Object task and d) the Recognition Memory task. Performance on these 4 tasks from Smithson Smithson and Gauthier (2025) was aggregated into an Om score

*Memory* *o* *tasks.* Four tasks required encoding and maintaining multiple object representations (Fig. 3). *Learning Exemplars with Vertical Ziggerins* involved memorizing six target objects presented together for 20 s, followed by alternating study-test phases (6, 18, then 24 recognition trials). Participants used “g,” “h,” and “j” keys to select which of three objects matched a memorized target. *Paired Objects* required learning five associations between novel 3D objects and abstract 2D shapes. Each pair appeared for 4 s with immediate testing, followed by multiple study-test cycles and 35 total test trials in various formats. *New Object* began with three novel objects (Quaddles) shown for 3000 ms, then presented 44 test arrays of four objects where participants identified the one that was novel (had never appeared before in the task). *Recognition Memory* involved studying successive sets of four target novel objects (15 s each) followed by a reading comprehension delay, then 24 recognition trials where targets appeared alongside two distractors from the same object category.

#### Control measures

*Working memory (WM)* was assessed using *Verbal-numerical Binding*, where participants remembered word-number pairs (2000 ms each, 1000 ms ISI) across sequences of 2–6 pairs, then identified correct pairings when probed with individual words or numbers (27 total responses). *Operation Span* required recalling letter sequences (1000 ms each) while performing intervening arithmetic operations (3000 ms or until response), with sequence recall tested after each trial (15 trials total).

*Low-level visual discrimination (LLV)* used the Hanover Early Vision Assessment (HEVA; Kieseler et al., 2022), presenting three images per trial (two identical, one different) across 96 trials. Participants pressed “f” (left), “space” (center), or “j” (right) to identify the odd stimulus. The battery comprised 16 blocks of six trials each, testing four basic visual features: dot distance (discriminating spacing between dot pairs), circle size (detecting size differences in circular stimuli), angle size (identifying angular differences in line configurations), and line length (distinguishing length variations in linear segments). Eight blocks were administered in session one and eight in session two, with each session containing two blocks per feature type.

*General intelligence (g)* was measured through *Raven’s Matrices* (selecting which of 8 numbered options completed 3 × 3 symbol patterns, 18 trials, 10-minute limit), *Number Series* (choosing the next number from 5 options, 15 trials, 5-minute limit), and a *Vocabulary* test (selecting the best synonym from 3 options, 30 trials, untimed).

#### Memory for spatial location, as a function of object meaningfulness

Object images in the location-memory task were adapted from Brady et al. (2008) and were selected from a large stimulus pool for which subjective ratings of stimulus familiarity (“How familiar is the stimulus?”) and stimulus knowledge (“Do you know what the stimulus is?”) were independently collected (Shoval et al., 2023b). In the original study, 198 participants rated 1,603 real-world objects; each participant was randomly assigned a subset of stimuli, with the constraint that at least 25 participants viewed and rated each image. Stimulus familiarity and stimulus knowledge were rated on a scale of 1–7. Because responses to the two questions were highly correlated (*r* =.98, *p* <.001), the ratings were averaged to form a single ‘meaningfulness’ measure, which was shown to predict memorability for both item identity (Shoval et al., 2023b) and item spatial location (Gronau et al., 2024).

**Fig. 4:**
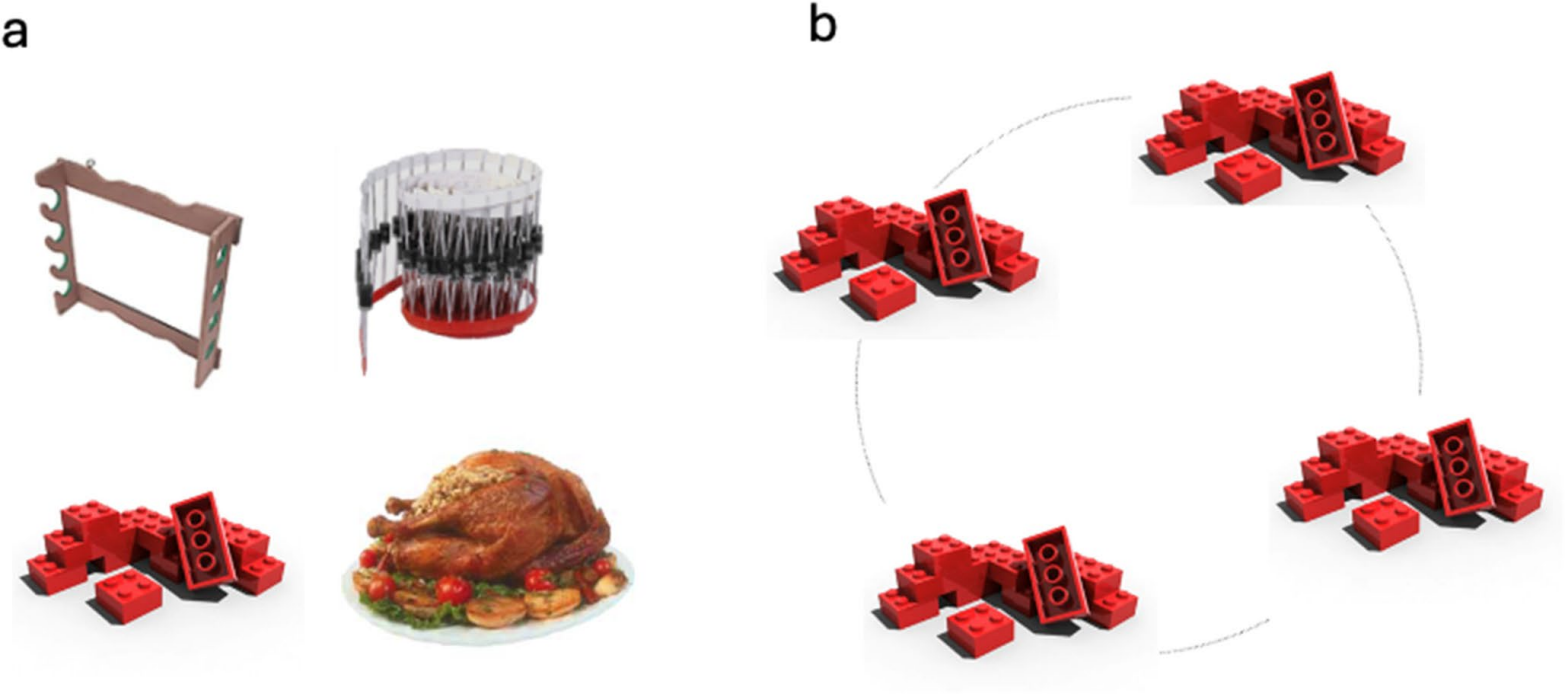
Episodic location memory task. **a** Example objects with low-meaning items on top, and high-meaning items on the bottom; **b** 4-AFC display testing for location memory of a specific object. Unbeknownst to participants, stimuli were displayed on an imaginary circle (light gray)

For the purposes of the present study, 288 objects were selected for the spatial location memory task which consisted of two phases: an encoding phase and a location-memory test phase. During the encoding phase, participants viewed the 288 object images, each presented individually for 2000 ms with an 800 ms inter-stimulus interval (ISI). The objects were positioned at one of 16 locations along the perimeter of an imaginary circle–unbeknownst to participants–with each point spaced 22.5° apart, starting at 11.25° and excluding the canonical axes. Each object spanned approximately 7 degrees, and the circle had a radius of about 11 degrees, assuming a viewing distance of 50 cm. However, as the experiment was conducted online, actual viewing distance may have varied across participants. Out of the 288 stimuli, 128 low-meaningful (average meaning score: 2.85, SD = 0.31) and 128 high-meaningful (average meaning score: 6.39, SD = 0.36) objects served as test items in the subsequent location-memory test phase (Fig. 4a). An additional 32 items (16 from each meaningfulness category) were repeated during the encoding phase and used in an item-repetition task (N-back, with repetition-lags of 0,1,2 or 4 items). These items only appeared during the encoding phase, ensuring maintenance of attention while viewing the images. Participants were instructed to memorize the spatial location of each item while simultaneously monitoring for item repetitions, responding by pressing the spacebar each time they detected a repeated item. The encoding phase included 320 trials in total, including item repetitions.

The memory-test phase involved a four-alternative forced-choice (4-AFC) test, in which each item appeared in its original location along with three additional locations positioned 90, 180, and 270 degrees from the encoded location. The four items in each trial were presented simultaneously until participants responded by clicking on the original location (Fig. 4b).

Note that object familiarity and level of recognition, which are combined to form our measure of meaningfulness, can reflect the richness of the semantic associative network linked to a well-recognized versus an unrecognized or poorly recognized object; however, this measure primarily captures an item’s level of familiarity. While such factors are known to influence memory (e.g., Dall et al., 2021; Reder et al., 2013), they are inherently subjective and likely shaped by multiple dimensions–including conceptual richness, typicality, nameability, and more. Despite this multidimensionality, the selected high-and low-meaning stimuli offered a promising starting point for investigating the relationship between *o*, semantic interpretation, and episodic location memory.

*Testing the reliability of the location memory test*. Before examining memory results and their correlation with *o* measures, it was important to assess the reliability of the episodic (location) memory measurements to ensure they were suitable for individual differences analyses. Note that paradigms that are sensitive at the group level do not always produce measurements that are sufficiently reliable for this purpose (Hedge et al., 2018; Ross et al., 2015). There are good reasons to believe that high reliability would be obtained for the measurements relevant to our first question – namely, whether people with a high *o* would remember object locations better. However, our second question – whether *o* would interact with the enhancement in location memory for meaningful versus less meaningful objects, is more challenging. Achieving reliability for the meaningfulness effect is likely to be more difficult because it involves comparing measurements obtained for the low and high meaningfulness conditions. In group-analyses, the meaningfulness effect would essentially be handled as a difference score between high- and low-meaningfulness items - but difference scores generally have low reliability (Hedge et al., 2018; Ross et al., 2015). Regressing out the variance for the low meaningful objects is a better option, although if performance on low- and high-meaningfulness items is highly correlated, the reliability of the residuals can still be low (Degutis et al., 2013; Ross et al., 2015). We therefore decided to perform item-level analyses on the memory scores to obtain acceptable reliability, before examining the correlation with *o.*

## Results

We considered possible self-selection biases for participants, out of those we have invited, who volunteered for this new study (*N* = 159), compared to those we did not hear back from (*N* = 96). There may be several reasons for whether a participant responded: The first study was conducted mid-August and the second mid-September, when some participants may have gone back to college. The two studies were conducted one month apart, with no explicit link between them, so it is unlikely that a participant would consider their experience in the first study (with no feedback on performance) when accepting the second one. The two groups (returning/not returning) did not differ significantly in gender distribution (returning 52% women; not returning, 56% women, χ²(1, 255) = 0.47 value, *p* =.49). Returning participants were on average 3 years older (33.7 vs. 30.6, t_253_ = 3.345, *p* <.001). There were no significant differences for *g* (t_253_ = 0.943, *p* =.35), working memory (t_253_ = 0.993, *p* =.32) or low level vision (t_253_ = 1.080, *p* =.28). Returning participants had on average a higher and less variable *o* (returning: mean = 0.38, SD = 0.33; not returning: mean = 0.27, SD = 0.42; Welch’s t_164_ = 2.22, *p* =.03; Levene’s test for equality of variances, F(1,253) = 6.586, *p* =.01). One possible reason why individuals with lower visual ability were less likely to participate in the second session is that the task description on Prolific mentioned “memorize the locations of different objects on the screen” whereas that for the first study was more vague: “you will complete a series of short tasks”. In any case, the reduced variability in *o* would most likely diminish its predictive power, suggesting that any effects we observe could be underestimated.

Table 1 includes descriptive statistics for each individual task, as well as for the composites included in our analyses. *Op* (*o* perception) and *Om* (*o* memory) are composite scores derived from each group of four tasks (equally weighted and normalized to z-scores), and *O* is the equally weighted composite of these two. WM, *g* and LLV (low-level visual ability) are also normalized, equally weighted composites of the 2, 3 and 4 tasks, respectively, used to estimate them. The reliability of average location judgments was very high (λ2 = 0.94) but that for the Meaningfulness effect was lower (λ2 = 0.55). The meaningfulness effect was operationalized as the advantage in location memory performance for high-meaning objects beyond what would be predicted based on performance with low-meaning objects. For each half of the data (odd/even trials from each condition), we regressed the high-meaning location memory performance on low-meaning location memory performance and extracted the residuals. The residuals from each half of the data were then correlated, and this correlation was corrected using the Spearman-Brown formula to estimate the reliability of the full-length meaningfulness effect measure.

**Table 1:** Descriptive statistics for individual tasks and composite variables

Task/Variable	Mean		SD	Skewness	Kurtosis	Reliability
Op Many Objects Oddball	71.8	%	9.7	-0.08	-0.68	.63
Op Silhouette Matching	58.2	%	13.1	-0.13	-0.53	.71
Op Ensemble Perception	55.8	deg	14.4	0.45	-0.20	.79
Op 3AFC Matching	69.8	%	10.5	-0.87	0.82	.71
Om Learning Exemplars	52.1	%	16.6	-0.26	-0.09	.85
Om Paired Objects	68.9	%	12.7	-0.22	-0.59	.73
Om New Object	77.2	%	10.0	-0.70	0.39	.71
Om Recognition Memory	76.6	%	15.6	-0.29	-0.95	.77
WM Binding	63.1	%	15.5	0.23	-0.57	.70
WM Ospan	73.9	%	16.6	-0.57	0.40	.87
g Ravens	48.9	%	23.5	0.09	-0.89	.81
g Number	70.3	%	22.8	-0.59	-0.54	.81
g Vocabulary	62.6	%	14.6	-0.49	0.10	.75
LLV Circle	76.8	%	12.0	-1.02	1.24	.66
LLV Line	79.2	%	12.0	-1.52	3.31	.68
LLV Angle	77.3	%	12.7	-1.28	1.94	.71
LLV Dot	74.3	%	9.6	-1.97	5.30	.68
Location Memory	43.3	%	11.1	0.76	0.42	.92
Meaning Effect	0		0.11	0.28	0.04	.71
Op	0		0.69	-0.66	0.40	.85
Om	0		0.68	-0.07	-0.62	.88
O	0		0.58	-0.39	0.06	.90
WM	0		0.80	-0.12	-0.05	.83
g	0		0.73	-0.35	-0.15	.87
LLV	0		0.81	-1.74	4.66	.88

Reliability is Guttman lambda2 for simple tasks and Location Memory, Spearman-Brown Corrected for Meaning effect and according to Mosier (1943) for composite variables

Importantly, our goal in this work is not to test the meaningfulness advantage in location memory, which has been reported previously (Gronau et al., 2024; see also Taevs et al., 2010; DeWitt et al., 2012), but rather to ask whether, if a meaningfulness advantage is present, it is related to *o*. As noted above, the robustness of an effect at the group level is somewhat independent of the reliability of the measure when comparing participants. In most individual-differences work, tests are honed for their psychometric properties–often by selecting items that contribute to high reliability–prior to their use in addressing research questions. However, when a new measure is used, as is the case here, purification of the measure through item analysis may be necessary to achieve the required reliability.

For the meaningfulness effect, which we measure using residuals, reliability may be low because of the expected strong correlation between location judgments for both high- and low-meaning items, which is independent of the mean difference between them (Degutis et al., 2013; Ross et al., 2015). Mean performance on location memory, with all items included, was significantly better for high-meaning than for low-meaning objects (M high =.498, SD =.14; M low =.370, SD =.11; *t* = 19.20, *p* <.001, d_z_=1.55, 95%CI [1.31, 1.78]). To improve the reliability of the meaningfulness effect, we used an item analysis to drop the 25 low meaning trials that most correlated with the average of the high meaning condition, and we dropped the 25 high meaning trials with the highest difference between correlation with average of low meaning and correlation with average of high meaning. This operationalizes the belief that both conditions share the same location memory effect, as based on ‘pure’ perceptual stimulus factors, and that the high meaning condition includes an additional facilitation from meaningfulness. In practice, it is likely that the meaningfulness effect was limited by the fact that meaningfulness estimates were from an independent set of raters, and some of the objects may have corresponded to different or just more variable levels of meaningfulness in this different sample. This trial selection (dropping about 20% of the trials) was entirely blind to any other consideration. Critically for the purpose of individual differences, every participant received a new high and low meaning condition score computed on the same set of trials. We arbitrarily chose 25 trials to drop from each of the meaningfulness conditions (maintaining 103 images in each), without exploring alternatives, as a compromise to keep a large number of trials in the data while dropping a sufficient number to hopefully make an improvement to reliability. After dropping these trials, the mean difference was comparable (M high = 0.512, SD = 0.15; M low = 0.354, SD = 0.09; *t* = 17.49, *p* <.001, d_z_=1.41, 95% CI [1.19, 1.63]) and if anything, a little smaller. As can be seen in Table 1, this process resulted in a meaningfulness effect reliability above 0.7 (the true reliability, when this selection is applied to a new sample, may be lower because of sampling error).^1^

Next we report the zero-order correlations between the location memory performance, the meaningfulness effect, age and gender as well as our various composite measures.

As shown in Table 2, there was a significant positive correlation between *o* and location memory (*r* =.482, *p* <.001, 95% CI [0.35, 0.595]), indicating a positive association between a perceptual and an episodic memory index. Furthermore, the correlation between *o* and location memory for low meaning objects was, *r* =.434, *p* <.001, 95% CI [0.296; 0.554] and for high meaning objects was *r* =.446, *p* <.001, 95% CI [0.309, 0.564]. These correlations (not presented in the table) were not significantly different from each other (Steigers z = 0.339, *p* =.734). The correlation between the Meaningfulness effect and *o*, however, was significant (*r* =.212, *p* =.008, 95% CI [0.056, 0.359]). To clarify, this analysis relates *o* to the residualized performance for high meaning objects, i.e., controlling for performance for low meaning objects. The result indicates that *o* shares unique variance with performance for the location of high meaning objects that is not shared with performance for the location of low meaning objects. However, performance in both conditions was also likely to be partly influenced by factors other than *o*, including those that relate to location memory such as age, WM, g and LLV (see Table 2).

**Table 2:** Pearson correlations between study variables

	Age	Gender	Op	Om	O	WM	g	LLV	Meaning effect
Gender (Women=1)	.102								
OP	.021	-.126							
OM	-.035	-.152	.413						
O	-.008	-.165	.842	.839					
WM	-.006	-.125	.066	.382	.265				
g	.014	-.079	.579	.476	.628	.255			
LLV	.002	.101	.539	.334	.520	.170	.503		
MeaningEffect	.120	.239	.160	.197	.212	.080	.122	.105	
LocationMemory	.199	.031	.373	.437	.482	.188	.382	.283	.506

Uncorrected r-thresholds for *N*=154 are r≥ .158, *p*<.05; r≥ .208 *p*<.01; r≥ .264, *p*<.001

Therefore, we tested our prediction that *o* is related to location memory, and particularly so for high-meaning objects, using hierarchical multiple regression to control for these other variables. We first predicted location memory performance based on age, gender, g, LLV, WM and added *o* in a second step. Since *o* was measured using four visual-perceptual and four memory-recognition tasks, following the approach of Smithson et al. (2025), we also examined whether any observed relationship between *o* and location memory would survive when *o* was estimated using only the perceptual tasks that involve no memory demands – that is, the *Op* composite (Table 3). Hence, in a second model, we predicted location memory performance based on *Op*, again, while controlling for all other factors.

**Table 3:** Multiple regression models predicting location memory, showing the incremental prediction of *o* (top), or *Op* alone (bottom)

Criterion: Location Memory (Model including *o*)					
	b	SE	t	p	Incr. R-squared
Age	.003	.001	2.746	.007	
Gender	.022	.016	1.352	.178	
g	.017	.014	1.235	.219	
LLV	-.002	.012	-.150	.881	
WM	.009	.010	.877	.382	
***o***	.080	.018	4.340	.000	**.090**
F = 10.22, *p*<.000, adj. R-squared =.294					
Criterion: Location Memory (Model including *Op*)					
	b	SE	t	p	Incr. R-squared
Age	.003	.001	2.547	.012	
Gender	.016	.017	.950	.344	
g	.030	.014	2.124	.035	
LLV	.003	.013	.244	.808	
WM	.018	.011	1.673	.097	
***Op***	.039	.016	2.484	.014	.**032**
F = 7.57, *p*<.000, adj. R-squared =.236					

Gender is coded as Women = 1, not-Women = 0

Both models were significant and, in both cases, there was a significant incremental contribution of *o* (9%) or *Op* alone (3.2%) to the explained variance in location memory performance. In addition, age was a significant predictor in both models, and *g* was a significant predictor only in the second model (likely because of the slightly higher correlation for *o* than *Op* with *g*).

**Fig. 5:**
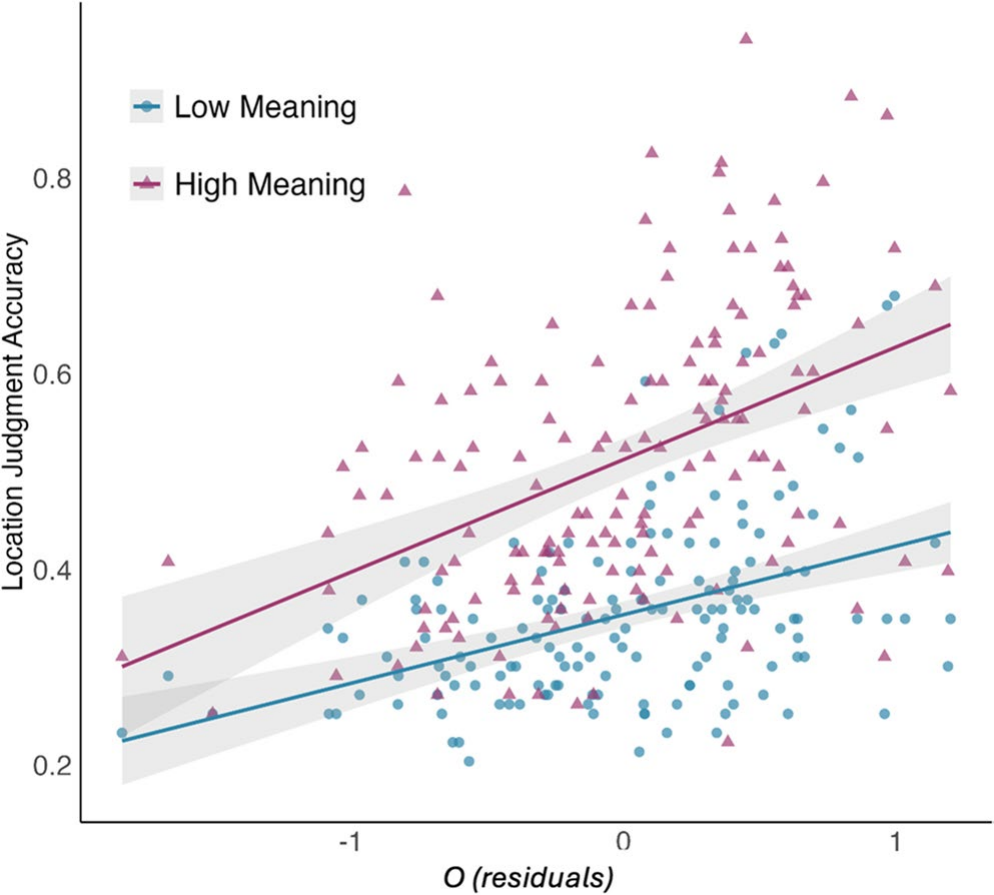
Semi-partial correlations between *o* (controlling for g, LLV, WM, age and gender) and location memory as a function of meaningfulness (with 95% C.I.). Low meaning: *r* =.246, high meaning: *r* =.341. Each participant has one datapoint in each meaningfulness condition

Next, we tested our prediction that *o* should be related to the meaningfulness effect with hierarchical multiple regression. In the first step we predicted location memory for high meaning objects based on age, gender, g, LLV, WM, and also controlled for location memory for low meaning objects, while in the second step we added *o* (or *Op* in a second model, see Table 4) as a predictor. Both models were significant and in both cases, there was a significant incremental contribution of *o* (3.2%) or *Op* (1.5%) to the explained variance in location memory performance. Gender was a significant predictor in both models, with women performing better. Figure 5 illustrates the semi-partial correlations between *o* (controlling for age, gender, LLV, WM and g) and location memory judgments for each condition. We use semi-partial correlations to show both the main effect of meaningfulness, and its interaction with *o*.

**Table 4:** Multiple regression models predicting location memory for high-meaning stimuli, showing the incremental prediction of *o* (top), or *Op* alone (bottom)

Criterion: Location for High Meaning (Model including *o*)							
	b	SE	t	p		Incr. R-squared	
Age	.002	.001	1.469	.144			
Gender	.064	.018	3.563	.000			
g	.000	.016	.003	.998			
LLV	-.011	.013	-.821	.413			
WM	.009	.011	.768	.444			
Low Meaning	.901	.105	8.583	.000			
*o*	.066	.021	3.105	.002		**.032**	
F = 22.47, *p*<.000, adj. R-squared =.519							
Criterion: Location for High Meaning (Model including *Op*)							
	b	SE	t	p		Incr. R-squared	
Age	.002	.001	1.279	.203			
Gender	.061	.018	3.332	.001			
g	.008	.016	.474	.636			
LLV	-.009	.014	-.635	.526			
WM	.016	.012	1.374	.172			
Low Meaning	.952	.104	9.121	.000			
*Op*	.036	.017	2.072	.040		**.015**	
F = 20.98, *p*<.000, adj. R-squared =.501							

Gender is coded as Women = 1, non-Women = 0

## Discussion

We aimed to assess the contribution of *o*, a domain-general skill reflecting the ability to recognize and distinguish visually similar items based on shape, to episodic memory, and specifically to memory for an object’s location. Even when manipulated arbitrarily and independently of an item’s identity, location memory can be positively influenced by a stimulus’s level of familiarity and/or semantic meaning (DeWitt et al., 2012; Gronau et al., 2024; Taevs et al., 2010). We therefore further examined whether *o* predicted the accuracy of location judgments and whether *o* interacted with stimulus meaningfulness to predict this aspect of episodic memory. Our findings provide empirical evidence for both questions. First, individuals with high *o* exhibited enhanced location memory compared to those with low *o*. Second, supporting a Perceptual-Semantic Synergy account, the relationship between *o* and location memory was stronger when participants encoded and retrieved high-meaningfulness items.

These findings provide the first direct evidence linking individual-level visual recognition skills with episodic (location) memory. In addition, our findings implicate semantic meaning, determined by the level of stimulus familiarity and identification, as a potential mediator of this relationship. *O* is related to**–**but clearly distinct from**–**a range of perceptual abilities (both visual and auditory), as well as higher-level cognitive functions such as working memory and general intelligence (Richler et al., 2019; Smithson et al., 2024; Sunday et al., 2017). Our results extend previous research by suggesting that enhanced visual encoding in individuals with high *o* contributes to improved memory for instance-specific details, such as object location. Notably, this effect remains robust even after accounting for other cognitive and low-level abilities. Additionally, we emphasize the facilitating role of item meaningfulness in this relationship. Each of these findings is discussed in detail below.

### High object recognition ability is linked to improved location memory

The ability to extract the invariant properties of an object, whether highly familiar or novel, is central to the *o* ability, which underlies robust item identification across varying viewing conditions. At the same time, our findings indicate that individuals with high *o* scores are also highly sensitive to contextual information that is extrinsic to the object itself, such as the screen location in which it was presented. Although these capabilities may seem contradictory **–** attending both to invariant features related to object shape and to a feature like location that can clearly vary without any influence on object identity **–** our results suggest they may nonetheless reflect shared underlying cognitive mechanisms. That is, the ability to encode both stable and context-dependent aspects of an object may represent complementary facets of a common cognitive process. Furthermore, given that spatial location is a well-established component of episodic memory **–** the “when” and “where” of an event **–** these findings may further blur the traditional boundaries between perception and memory.

Our correlational design does not allow us to distinguish item memory and item-location binding effects, as in paradigms where both features and locations are unique (e.g., Chalfonte & Johnson, 1996). In our paradigm, remembering the specific location of an item largely relies on accurate encoding of the item itself, as well as successful item-location binding **–** both processes that may be more efficient in individuals with high *o* scores. Our findings are also consistent with models emphasizing the role of attentional and working memory resources, which propose that efficient item encoding frees up cognitive capacity for binding the items to their contextual surroundings (Popov & Reder, 2020; Reder et al., 2013).

Several researchers have suggested that spatial location, along with processes related to location-identity binding, may benefit from a unique attentional and memory status (e.g., Golomb et al., 2014; Hollingworth, 2006; Kovacs & Harris, 2019; Treisman & Gelade, 1980). This is reflected in findings showing that not only do objects serve as effective memory cues for their encoded locations, but locations can also cue item identity and the precise visual details of objects. For instance, participants more accurately recognized specific object exemplars and episodic details (such as pose) when items were tested at their original location, compared to when they were presented at a different location in the scene (Hollingworth, 2006). However, this enhanced item discrimination due to shared location may come at a cost. When successive pairs of identical or different objects were presented in the same location, participants were biased to report that the identities were the same, even when they were not (Golomb et al., 2014; Pertzov & Husain, 2014). In other words, shared location increases both correct identifications (hits) and false alarms, suggesting that participants are unable to disregard spatial overlap between stimuli, even when it is irrelevant and detrimental to the task. Although our task involved a long-term memory paradigm, many stimuli appeared in repeated locations, which may have introduced interference in location memory. Nevertheless, high-*o* participants appear to benefit from enhanced item identification and distinctiveness, which may help mitigate such location-based interference.

This pattern suggests that high *o* supports more precise object-location bindings by enhancing the fidelity of object encoding and reducing confusion between overlapping spatial cues. Our findings echo prior work showing that memorable images aid spatial recall (Trinkl & Wolfe, 2024) – in that work, the effect was specific to memory for spatial location, not extending to temporal position in the studied sequence. Interestingly, other work (Smithson et al., 2025) found a strong correlation between *o* and visual spatial ability–that is, the capacity to perceive, process, and mentally manipulate visual material (as measured by mental rotation tasks; Schneider & McGrew, 2018). However, although both location memory and mental rotation involve spatial processing, they may depend on qualitatively different cognitive mechanisms. Notably, the relationship between *o* and spatial ability was largely accounted for by their shared association with general intelligence (Smithson et al., 2025). Here, the contribution of *o* to location memory remained robust even after statistically controlling for general intelligence and working memory and was still obtained when using only perceptual *o* tasks. This strengthens the idea that *o* facilitates the encoding of spatial information because of mechanisms supporting high-level visual processing, rather than associated cognitive abilities.

From a neurocognitive perspective, one theory that aligns with our findings and bridges visual perception and episodic memory is the representational-hierarchical model (Bussey et al., 2002). This model proposes that the ventral visual stream builds increasingly integrated object representations, with the perirhinal cortex (PRC) supporting complex, feature-rich object codes (Barense et al., 2007; Li et al., 2022). Episodic memory mechanisms, including those in the hippocampus, operate on these PRC-level representations to bind objects to their spatial and contextual relations. Our behavioral findings align with this representational logic: individuals with higher *o* may encode objects with greater distinctiveness, thereby providing more effective input for item–location binding. Importantly, the link to this model is theoretical rather than anatomical. We raise it to illustrate how, within this framework, perceptual and episodic processes may relate through shared representational demands, offering a coherent explanation for why a perceptual ability and an episodic outcome are empirically associated.

### High object recognition ability predicts the meaningfulness effect on location memory

One of the central findings of our research is that the semantic meaning of objects moderates the relationship between *o* and location memory. In prior work, we found that *o* measured with sets of tasks that use both familiar (real-world) or artificial novel objects is essentially the same at the latent level (Sunday et al., 2021). Nevertheless, here *o* interacted with object’s meaning in the context of location memory, where stimulus ‘meaningfulness’ corresponds to its level of knowledge and familiarity. Specifically, a stronger positive correlation emerged between *o* and location memory judgments for high-meaning objects compared to low-meaning ones. As noted earlier, we interpret this effect as reflecting a more efficient use of semantic knowledge embedded within meaningful objects by individuals with high *o*, consistent with prior evidence that such individuals demonstrate enhanced ability in subordinate-level discriminations (Sunday et al., 2021). When an object is recognized and encoded at a more refined level, it can activate a broader semantic network and carry greater personal relevance. Indeed, higher expertise is linked to the ability to make finer subordinate-level distinctions, which tend to carry rich semantic content (Johnson & Eilers, 1998). Furthermore, experts demonstrate superior short- and long-term memory within their domain, likely due to the combined effects of visual and conceptual training (e.g., Annis & Palmeri, 2019; Curby & Gauthier, 2007; Herzmann & Curran, 2011).

The role of semantic knowledge in long-term memory formation and retrieval has long been established (e.g., Anderson, 1984a, b; Brewer & Treyens, 1981; Craik & Tulving, 1975). However, one line of research has brought this topic back to the forefront, particularly in the context of memory for specific visual details (e.g., Brady et al., 2008). For example, Konkle et al. (2010) found that interference in visual long-term memory is primarily predicted by conceptual distinctiveness rather than perceptual distinctiveness among similar object exemplars. More recently, Kramer et al. (2023) demonstrated that semantic features have a stronger influence than perceptual features on the memorability of objects and scenes. According to one account, highly memorable images may be more easily recognized *because* they are more effectively mapped onto semantic features (Deng et al., 2024). Additionally, visual working memory capacity is higher for meaningful objects than for meaningless items or simple features, likely due to semantic associations rather than visual complexity alone (Shoval et al., 2023b; Torres et al., 2025).

Semantic meaning can enhance memory for both intrinsic object properties such as color (Chung et al., 2023; Gronau & Shachar, 2015) and extrinsic details, such as the object’s location or scene background (Gronau et al., 2024; Reder et al., 2013; Sahar et al., 2024). Conceptual meaning has been viewed as a “hook” or “scaffold” that supports the retention of perceptual details in memory (Chung et al., 2023; Konkle et al., 2010). While such metaphors offer useful heuristics, other researchers have sought to clarify the underlying mechanisms by which highly familiar items benefit memory more than novel or meaningless stimuli. As discussed earlier, resource-limited theories propose that familiar items place lower demands on working memory during encoding, thereby freeing up resources to store visual details and bind items to their contextual surroundings (Popov & Reder, 2020; Reder et al., 2013). But the impact of semantic meaning extends beyond simple familiarity, reflecting a richer interplay of conceptual knowledge, categorical specificity, and/or verbal encoding processes. In particular, verbal labeling has been proposed as one of several mechanisms underlying the memory advantage for meaningful over abstract or unfamiliar objects, including their enhanced recall in location memory tasks (Choi & L’Hirondelle, 2005; Taevs et al., 2010). In Choi and L’Hirondelle (2005), women remembered the location of objects better than men when the objects were concrete rather than abstract. Thus, verbal processing may also help account for the gender difference observed in the present study: women demonstrated greater location memory benefits from object meaningfulness than men. This finding aligns with well-established evidence of female advantages in verbal processing (Bleecker et al., 1988; Kramer et al., 2023), suggesting that stimulus-naming strategies could have contributed to the observed female advantage in object-location memory tasks. However, it appears unlikely that a useful verbal strategy would involve encoding both the name of the object and a verbal description of its location for most stimuli; the relatively large number of stimuli, as well as the absence of ‘canonical’ locations (e.g., right–left or up–down, positioned at 0/180 or 90/270 degrees), would make this relatively difficult. Moreover, given that the memory test employed a four-alternative forced-choice format, verbalizing a single spatial dimension (e.g., “up” or “right”) would not uniquely specify the object’s location, as each spatial label was shared by at least two stimuli. An alternative is that a better ability to name stimuli during encoding would support stronger semantic processing, which we believe supports richer episodic encoding. This should be explored in future studies. Importantly, we cannot entirely rule out the possibility that residual verbal processing contributed to the meaningfulness effect, and specifically to the observed gender effect, but this would be independent of the relationship we observed between *o* and location memory, as *o* tasks do not encourage verbal strategies and *o* is not related to gender.

There are some limitations in this work. We tested participants from a prior study and were therefore constrained both in terms of sample size (by participants who willingly signed up for this follow-up) and in terms of the covariates that were included. Our study focused on one aspect of episodic memory, namely, spatial location, which may not fully represent other components of episodic memory, such as temporal or other contextual factors. As a result, the generalizability of our findings may be limited.

Furthermore, we used a coarse measure of meaningfulness, characterized by raters’ understanding of what that concept means, and which likely conflates several dimensions such as familiarity, nameability and complexity. Therefore, this work cannot specify which aspects of meaningfulness may be responsible for interacting with object-location memory. Moreover, our meaningful objects were more likely to be living (e.g., animals or food), whereas the low-meaning items were mainly inanimate tools and objects, which differed in some visual properties from the high-meaning objects. A low-level feature analysis of the full stimulus set revealed that high-meaning stimuli were more colorful, exhibited greater color variability, and tended to have natural or matte surface properties. In contrast, low-meaning objects were characterized by more neutral, desaturated, and brighter colors, consistent with the industrial or mechanical materials represented in some of these items. The two meaning conditions were closely matched for other properties such as shape and object size (see *Supplemental Material* for the full analysis). The differences found in low-level features between the two categories are not surprising, given that categories of natural images often differ in their low- and mid-level statistics depending on the semantic content of the images (e.g., Long et al., 2016; Torralba & Oliva, 2003). Thus, the meaningfulness effect may, in part, be influenced by differences in low-level visual features, particularly color, between the high- and low-meaning object sets. Critically, these findings do not preclude contributions from semantic meaning or familiarity. Low-level object statistics and high-level item knowledge are not mutually exclusive; rather, both may jointly influence object-location memory and the observed meaningfulness effect. Indeed, neural evidence indicates that sensitivity to differences in low-level features, as represented in early visual cortex, can directly support the construction of high-level semantic and categorical representations, whereby the visual system leverages low-level statistical regularities to compute category structure (e.g., Henderson et al., 2023). Such findings apply to a variety of features, including color specifically as a property utilized for distinguishing between animate versus inanimate objects (Rosenthal et al., 2018). Thus, it appears that the visual differences observed between the two meaning categories may affect performance at lower levels of processing, while also interacting with, and supposedly amplifying, higher-level semantic and familiarity-based processes.

In sum, our results demonstrate that mechanisms that support a better domain-general object recognition ability, *o*, also play a role in episodic memory by enhancing the binding of meaningful visual objects to spatial context. This challenges traditional cognitive boundaries, showing that perceptual skills contribute to memory encoding processes. Even after controlling for intelligence, working memory, and low-level visual abilities, *o* remains a significant predictor of performance. The influence of meaningfulness as a moderator highlights the pivotal role of semantic content and stimulus familiarity in memory formation. Our work opens the door to further investigations into the perceptual and semantic underpinnings of episodic recall. These findings call for models that integrate visual and semantic processing to explain how meaning facilitates memory.

## Supplementary Information

Below is the link to the electronic supplementary material.

Supplementary Material

## Data Availability

All experimental materials and results are available at the Open Science Framework and can beaccessed at [https://osf.io/hy763/?view\_only=be6bc532f4fa490d98e81e7eb1d8eb86](https:/osf.io/hy763/?view_only=be6bc532f4fa490d98e81e7eb1d8eb86). This study was not preregistered.

## Footnotes

1 Of the four critical multiple regressions reported in Tables 3 and 4, all are statistically significant when all trials are included, except for Op predicting the meaningfulness effect, which is the smallest of our effects (*p* =.378). Therefore, it appears that using the more reliable measure increases power but does not otherwise qualitatively change the nature of the effects.
