# Supplementary material for: Object recognition ability predicts episodic location memory, enhanced by meaningfulness
Source: Psychol Res. 2026 Feb 7;90(1):22. doi: 10.1007/s00426-026-02248-y (PMC12881127; doi:10.1007/s00426-026-02248-y)
Supplement: Supplementary file 1 — Supplementary Material [file 426_2026_2248_MOESM1_ESM.pdf]

Supplementary Material: Low-Level Visual Feature Comparison Analysis

S1. Methods

We analyzed 256 images across two conditions: Low-meaning (n = 128) and High-meaning (n = 128). Twenty-two low-level visual features were extracted from each image, organized into four categories: spatial structure, color, luminance and texture, and object size.

S2. Feature Definitions

S2.1 Spatial Structure

edge_density: Mean Canny edge detector response.

fft_low: Low spatial frequencies (0–0.15); reflects coarse structure and overall shape.

fft_mid: Mid spatial frequencies (0.15–0.4); reflects object boundaries and medium-scale features.

fft_high: High spatial frequencies (0.4–1.0); reflects fine details, textures, and edges.

fft_low_high_ratio: Ratio of low to high frequency energy. High values indicate energy concentrated in coarse structure (simple, canonical shapes); low values indicate energy in fine details (complex, fragmented forms).

fft_mid_high_ratio: Ratio of mid to high frequency energy. High values indicate energy in boundaries (coherent contours); low values indicate energy in textures (cluttered, mechanical details).

S2.2 Color

mean_sat: Mean HSV saturation (0 = grayscale, 1 = fully saturated).

sat_range: Range of saturation values. Low values indicate a narrow palette (metallic, neutral); high values indicate a broad palette (natural, diverse colors).

sat_sd: Standard deviation of saturation. Low values indicate uniform color appearance; high values indicate varied color palette.

hue_var: Variance of hue values (color diversity).

rg_mean: Mean red-green opponent channel (positive = reddish, negative = greenish).

rg_range: Range of red-green variation (chromatic modulation).

by_mean: Mean blue-yellow opponent channel (positive = bluish, negative = yellowish).

by_range: Range of blue-yellow variation (chromatic modulation).

S2.3 Luminance and Texture

lum_contrast: Standard deviation of grayscale values.

lum_skewness: Asymmetry of luminance distribution. Positive values indicate bright tail (specular highlights); negative values indicate dark tail (shadows); near-zero indicates symmetric distribution.

lum_kurtosis: Tailedness of luminance distribution. High positive values indicate extreme values (high gloss, strong specular reflections); near-zero indicates normal distribution; negative values indicate flat distribution.

texture_entropy: Shannon entropy of grayscale histogram (texture complexity).

symmetry: Horizontal mirror correlation (bilateral symmetry; range: −1 to 1).

S2.4 Object Size

object_area_prop: Proportion of non-white pixels. Assumes light background; range 0–1.

object_area_otsu: Proportion using automatic (mean intensity) threshold. More robust to varying background brightness.

elongation: Aspect ratio of object's spatial extent calculated from second moments. Value of 1 = circular/square; >1 = elongated.

S3. Descriptive Statistics

Table S1 presents means and standard deviations for all features by condition.

Table S1. *Descriptive statistics for low-level visual features by condition.*

	Low-meaning		High-meaning	
**Feature**	**M**	**SD**	**M**	**SD**
edge_density	0.020	0.009	0.021	0.009
fft_low	7.11	8.72	7.32	9.49
fft_mid	17.7	18.8	18.0	18.3
fft_high	68302	10645	66705	12931
fft_low_high_ratio	0.000114	0.000158	0.000114	0.000149
fft_mid_high_ratio	0.000283	0.000338	0.000283	0.000281
mean_sat	0.087	0.072	0.131	0.093
sat_range	0.958	0.184	0.986	0.075
sat_sd	0.156	0.088	0.206	0.095
hue_var	0.045	0.037	0.049	0.039
rg_mean	0.015	0.033	0.029	0.055
rg_range	0.405	0.278	0.583	0.353
by_mean	-0.022	0.050	-0.036	0.054
by_range	0.484	0.302	0.602	0.313
lum_contrast	0.244	0.074	0.258	0.075
lum_skewness	-1.79	1.25	-1.46	1.11
lum_kurtosis	3.69	7.07	2.16	6.74
texture_entropy	2.43	0.992	2.63	0.901
symmetry	0.346	0.241	0.385	0.256

object_area_prop	0.308	0.169	0.335	0.153
object_area_otsu	0.285	0.137	0.312	0.126
elongation	1.90	1.07	1.75	0.764

Note. Low-meaning n = 128; High-meaning n = 128.

S4. Statistical Comparisons

Table S2 presents Wilcoxon rank-sum test results comparing Low-meaning and High-meaning conditions for each feature.

Table S2. *Wilcoxon rank-sum tests comparing conditions.*

Feature	W	p	
edge_density	7298	.132	
fft_low	8275	.889	
fft_mid	7989	.733	
fft_high	9149	.106	
fft_low_high_ratio	8089	.863	
fft_mid_high_ratio	7792	.500	
mean_sat	5730	< .001	***
sat_range	7861	.279	
sat_sd	5832	< .001	***
hue_var	7711	.417	
rg_mean	6645	.009	**
rg_range	5573	< .001	***
by_mean	9811	.006	**
by_range	6231	< .001	***
lum_contrast	7327	.145	
lum_skewness	6967	.039	*
lum_kurtosis	9574	.020	*
texture_entropy	7261	.116	
symmetry	7432	.200	
object_area_prop	7343	.152	
object_area_otsu	7264	.117	
elongation	8685	.406	

Note. * $p < .05$, ** $p < .01$, *** $p < .001$

S5. Summary of Significant Differences

Eight features showed significant differences between conditions (Table S3). All significant differences were in color and luminance features; no spatial structure or object size features differed between conditions.

Table S3. *Features with significant differences between conditions.*

Feature	Direction	% Difference	p
rg_range	Lower in Low-meaning	30.6%	< .001
mean_sat	Lower in Low-meaning	33.3%	< .001
sat_sd	Lower in Low-meaning	24.1%	< .001
by_range	Lower in Low-meaning	19.6%	< .001
by_mean	Higher in Low-meaning	37.9%	.006
rg_mean	Lower in Low-meaning	49.9%	.009
lum_kurtosis	Higher in Low-meaning	71.1%	.020
lum_skewness	Lower in Low-meaning	22.4%	.039

Note. *Ordered by significance level.*
